# An Adaptive Algorithm for Multipath Mitigation in GNSS Positioning with Android Smartphones

**DOI:** 10.3390/s22155790

**Published:** 2022-08-03

**Authors:** Lorenzo Benvenuto, Tiziano Cosso, Giorgio Delzanno

**Affiliations:** 1 DIBRIS—Department of Informatics, Bioengineering, Robotics and Systems Engineering, University of Genoa, 16146 Genoa, Italy; lorenzo.benvenuto@edu.unige.it; 2 Gter s.r.l, 16128 Genoa, Italy; tiziano.cosso@gter.it

**Keywords:** GNSS positioning, data analysis, internet of things, mobile computing

## Abstract

We present a solution for improving the robustness of GNSS positioning with Android devices. The proposed method combines an acquisition phase performed in a dedicated Android app (thus working on the edge) and a processing phase, based on a modified version of the open source library RTKLIB, performed on a dedicated server. The processing phase applies an improved version of the RTK library based on an adaptive algorithm for mitigating the multipath effect on satellite radio signals received by smartphone’s antennas. The algorithm is built on top of an extended version of the sigma-epsilon model in which weights associated to observables potentially affected by multipath errors are computed using logged data. In the paper, we will focus our attention on the architecture of the proposed solution and discuss preliminary experimental results obtained with the resulting system.

## 1. Introduction

### 1.1. Background and Motivations

In May 2016, during the “Google I/O” conference, Google released an API to give Android developers access to GNSS raw measurements such as carrier phase, code measurements, and navigation messages. As stated in the white paper [1] of the GNSS Raw Measurement Task Force, coordinated by the European GNSS Agency (GSA), this new feature of the Android API offered new research directions. In particular, GNSS raw measurements can be used to optimise multi-GNSS and multi-frequency solutions, to select satellites based on their performance, to transfer processing techniques from GNSS receivers to smartphones, to combine GNSS raw data with data of other sensors that are available in smartphones, and to enable testing and post-processing analysis [2,3,4,5].

From a technical point of view, the use of GNSS raw measurements posed several challenges for both GNSS experts and software developers. Indeed, on one hand, GNSS standard formats, such as RINEX or NMEA, are not natively available on the Android platform. On the other hand, mobile app developers are not generally familiar with the complex algorithms and libraries used in GNSS positioning.

The GSA white paper [1] addressed the gap between the two fields, providing useful information, for example, for deriving the pseudoranges from Android. This important work opened a new research field aimed at developing low-cost applications for satellite-based positioning systems. In particular, after its publication, many authors started to analyse the quality of the raw measurements retrieved from smartphones and compare them with other types of low-cost devices. The main detected issue turned out to be the high noise of the GNSS observables. Indeed, smartphones are equipped with cellphone-grade GNSS chipsets and antennas, which have on average very low gain, resulting in a low and irregular signal-to-noise ratio (SNR) [5]. For this reason, smartphone positioning is very challenging, especially in harsh environments, such as urban areas, that are more vulnerable to multipath and other interferences (see e.g., [6,7,8,9,10,11,12,13]). Other issues were related to the duty cycle mechanism and to low values of the C/N_0_ [14,15].

In [16], the authors demonstrated that it was possible to reach decimetric accuracy in terms of positioning performances following the post-processing approach, via a double difference of raw smartphone observations. Meanwhile, the authors of [17] first focused their attention on single-base RTK positioning and then demonstrated the possibility of obtaining centimetre-level accuracy through the use of NRTK corrections [17]. These results are supported by the authors in [18], who, employing a variometric approach, show decimetre accuracy in static conditions and sub-metre when used in an urban vehicle scenario. Very interesting and promising results have also been obtained by applying PPP (precise point positioning) techniques to smartphones devices. In [19], the authors analysed single-frequency PPP in static mode and reported that decimetre- to meter-level positioning accuracy can be achieved with the smartphone-grade hardware, while in [20], a smartphone software application called PPP WizLite is proposed. This application enacts PPP processing on smartphones using Doppler-smoothed code pseudoranges. The authors in [20] also showed that positioning accuracies at the sub-metre and metre level can be achieved in static and kinematic mode, respectively. In [21], the authors developed a new methodology for the PPP processing in smart devices, achieving sub-meter-level positioning accuracy. In particular, they wrote a software application based on the Android platform, named Smart-PPP, that implements a modified stochastic model in PPP processing to weight code and carrier phase measurements on the basis of SNR parameter. To achieve this goal, an improved uncombined PPP observation model is proposed in Smart-PPP. A modified C/N0-dependent weighting strategy is employed. Some data-processing strategies used in classical PPP with the geodetic receiver are improved and made more suitable for the data characteristics of smart devices. By applying the Smart-PPP approach to smart devices, the final positioning results can be smoother and more accurate.

Another milestone has been set by Broadcom, which announced, on 21 September 2017, the world’s first mass-market, dual-frequency GNSS receiver device, the BCM47755. In May 2018, the Xiaomi Mi8 (Mi8) became the first smartphone in the world, employing a dual-frequency GNSS receiver L1/E1–L5/E5a. This led to the next series of studies in the investigation of smartphone-based positioning. Thanks to the double frequency introduced in [22], the multipath performance of the Xiaomi Mi8 device was investigated for both E1/L1 E5a/L5 signals using a proper linear combination. The results obtained were quite promising, but they also seem to indicate multipath as one of the main problems for smartphone positioning. Multipath effects on Android devices were also studied in [23], where the author performed positioning with the Nexus 9 tablet using a particular Eccosorb for multipath mitigation. The results shows that precise positioning with uncertainties lower than one metre was possible. In [24], the authors show encouraging results in ZTD estimation by smartphone devices.

Nowadays, raw GNSS measurements support is mandatory on devices that run Android 10 (API level 29) or higher, but unfortunately, the support for some of the raw GNSS measurement fields (e.g., pseudorange rate, ADR, AGC) is optional and can vary based on the type of GNSS chipset installed on the device. Furthermore, not all the smartphones present on the market support double frequency or multi-constellation [25]. For this reason, finding a robust use of GNSS raw measurements is still a topic of interest for the research communities working on GNSS and mobile computing.

### 1.2. Research Question

As mentioned before, the multipath effect, i.e., radio signals reaching the receiving antenna by two or more paths, is probably the major source of error in urban scenarios affecting the positioning quality. A series of tests carried out with smartphones equipped with dual-frequency receivers (Broadcom and Snapdragon chipsets) confirmed this hypothesis experimentally. Multipath is also a serious problem for the application of GNSS positioning algorithms that use raw measurements.

Our research question is whether multipath mitigation techniques used for GNSS receivers can be applied to RTK positioning with Android devices. RTK positioning is a class of algorithms that employ correction codes received from base stations. They are particularly interesting since they can reach centimetric accuracy without the need of positioning information from cellular and Internet networks. In particular, in this setting, our goal is to investigate different types of heuristics to increase robustness, in terms of precision and accuracy, of the RTK positioning in Android devices.

### 1.3. Our Contribution

Our first contribution is the design and implementation of a prototype system for applying multipath mitigation heuristics in RTK positioning with GNSS raw measurements. The proposed system is based on a pre-processing phase performed in a dedicated Android app (thus working on the edge) and on a real-time processing phase, based on a modified version of the open source library RTKLIB [26], performed on a dedicated server. The performance of the resulting system, including client-server latencies, is comparable to RTK positioning procedures for GNSS receivers and assisted GPS computing procedure. It is important to note that both other solutions also require network communication steps. The data acquisition phase, performed via an Android app developed during Lorenzo Benvenuto’s PhD work [27], is aimed at cleaning, filtering, organizing, and delivering the data acquired via the GNSS raw measurements library. The app is in charge of collecting the satellite data of a given epoch, namely raw observations of different satellites and frequencies, converting them into a special message format, and sending the resulting message to the processing server. The app has several other functionalities, including that of receiving and visualizing the position inferred by our algorithms and providing options to control the acquisition phase (e.g., flags to enable/disable duty cycle and real-time plot of the SNR parameter).

The server-side processing phase exploits an improved version of the RTKLIB library, enabling RTK positioning from smartphones and increasing the solution robustness by means of multipath mitigation heuristics. In order to make RTKLIB work in real time with smartphones, a new API for processing GNSS data in the format defined for data collection was added to the original library. Concerning the multipath mitigation, the so-called MDP (multipath detection parameter) algorithm, conceived and patented by Gter, was implemented in the RTKLIB version adopted in this work. This algorithm performs multipath detection and mitigation in real time for single-frequency GNSS receivers. The MDP algorithm was improved and adapted for working with GNSS observables from Android devices. The main idea here is to weigh observations of each piece of satellite data with parameters associated to multipath (MDP variable) and signal noise (SNR) errors. The resulting weights are used to tune the RTKLIB positioning algorithms in order to assign low weights to unreliable observations. The algorithm has several possible configuration parameters and operating modes. In particular, one of the biggest improvements of our algorithms is in the adoption of adaptive thresholds that are inferred via statistical analysis of collected data.

The procedure described above was tested and validated using two different data sets: a static acquisition with multipath effect induced and a kinematic acquisition. For both case studies, several combinations of the MDP algorithm configuration parameters were tested. The results obtained seem very promising. Indeed, with the proposed algorithm, improvements in positioning accuracy were noted, especially for the period of induced multipath, meaning that the MDP algorithm is suitable for the mitigation of this effect. Furthermore, in the solution obtained with the MDP algorithm application, some positioning outliers are eliminated, and consequently, the solution’s robustness is increased.

### 1.4. Originality and Reproducibility

The paper includes original work extracted from Lorenzo Benvenuto’s PhD thesis [27], supervised by Tiziano Cosso and Giorgio Delzanno. The source code for the GNNS Base station server and the modified version of RTKLIB presented in this paper are available on GitHub (https://github.com/gtergeomatica/RTKLIB_Android, accessed on 7 February 2022). See also [27].

### 1.5. Plan of the Paper

In Section 2, we present a preliminary comparison between smartphones and low-cost GNSS receivers. In Section 3, we present the IoT RTK system In Section 4, we present the MDP multipath mitigation algorithm. In Section 5, we discuss experimental results obtained on static and kinetic tests. Finally, in Section 6, we address some conclusions and discuss future directions for our work.

## 2. Positioning Analysis with Android Devices

In this section, we present an analysis of the signal quality of smartphone receivers and compare it with that of geodetic and GNSS receivers.

The device considered in all tests is the Xiaomi Mi 8. This smartphone is equipped with the Broadcom 47755 dual-frequency GNSS chip capable of tracking GPS L1 C/A, GLONASS L1, BeiDou B1, Galileo E1, GPS L5, and Galileo E5a. The device has been chosen as it can provide both pseudo-range and carrier phase measurements, and navigation messages. It is worth mentioning that not all the smartphones on the market have access to all the GNSS raw measurements.

We consider here two types of tests: kinematic acquisition and static acquisition with multipath induced. The setup of each test is described in detail in the rest of the section.

### 2.1. Test 1: Kinematic Acquisition

**Figure 1:**
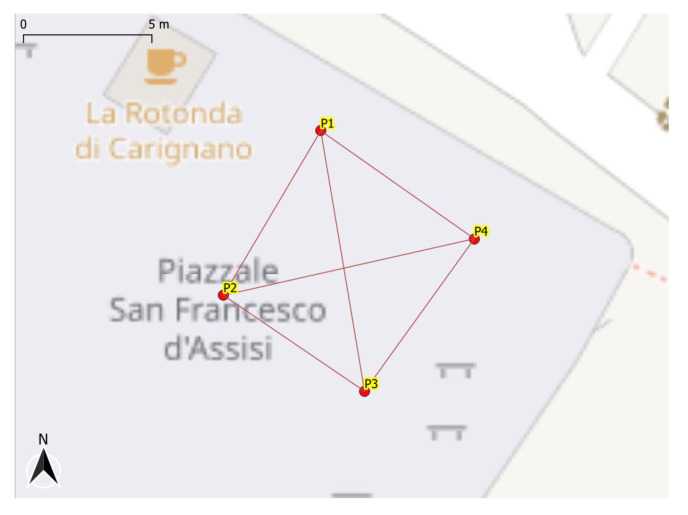
Trajectory used for test 1.

Navigation is one of the main areas in which the GNSS receiver embedded into a smartphone is employed. Test 1 aims at making an assessment of the performance of the GNSS positioning from a smartphone in a typical scenarios such as pedestrian navigation. More specifically, test 1 consists of a kinematic pedestrian acquisition involving two GNSS receivers, the Xiaomi Mi 8 and the Stonex S500, used for comparison. The Stonex S500 is a single-frequency (L1) and multi-constellation (GPS, GLONASS, Galileo, and BeiDou) device typically used for GIS (geographic information system) and RTK applications based on a u−blox chipset. The test path, which was located in Genoa and is shown in the Figure 1, was identified by determining the vertices of a square. In order to get the vertexes’ coordinates with high precision, the four vertexes were surveyed in NRTK modality with the Stonex S70G, a geodetic-level GNSS receiver, and exploiting the Ligurian NRTK network for getting the differential corrections. Once surveyed, the vertexes were materialised using four targets.

Test 1 started at vertex number 1 with a static acquisition of 2 min. After that, a walk between the four vertexes was performed along the squared sides, as depicted in Figure 1. Each side has been traversed in both directions (e.g., from vertex 1 to vertex 2 and from vertex 2 to vertex 1). The test ended at vertex 1 with a static acquisition of 2 min. Data from both the receivers were collected at 1 Hz rate. During the test, the two receivers were held at a height of about 1 m above the ground.

### 2.2. Test 2: Multipath Effect

Multipath and other interferences are one of the main sources of error in GNSS positioning in urban canyons, especially if mass market GNSS receivers are employed. Based on this consideration, test 4 aims at evaluating the impact of multipath in smartphone positioning.

**Figure 2:**
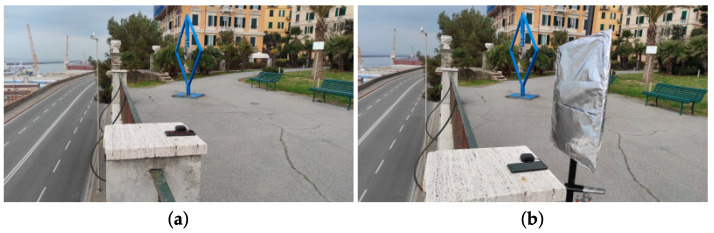
Test 2 acquisition under standard condition (**a**), and with multipath induced (**b**).

Similarly to test 1, a second GNSS receiver is used for comparing positioning results. Test 2 consists of static acquisition, in which, for a specific interval, the multipath effect was reproduced by placing a metal plate behind the receivers (see Figure 2). The receivers used in this test are the Xiaomi Mi 8, and the u−blox ZED F9P coupled with the AN-MB-00 patch antenna. The u−blox ZED F9P is a mass market multi-frequency (L1/L2) and multi-constellation (GPS, GLONASS, Galileo, and BeiDou) GNSS receiver.

The two receivers were placed at a point whose coordinates were determined with high precision in NRTK modality using the Stonex S70G receiver, and exploiting the Ligurian NRTK network for the differential corrections. The static acquisition started at 9:15 a.m. UTC time and lasted 1 h. From 9:45 to 10:00, the multipath effect was induced by placing the metal plate behind the receivers (see Figure 2). After 10:00, the plate was removed, and the acquisition ended at 10:15. Data from both the receivers were collected at 1 Hz rate.

### 2.3. Precision and Accuracy

Considerations regarding the accuracy and precision of the analysis of the acquired data sets can be made through the standard deviation (*STD*) and root mean square (*RMS*) values. Given a set of elements, *STD* and *RMS* can be defined as:

(1) $$\begin{array}{c} STD=\sqrt{\frac{1}{N}\;\cdot \;\sum_{k=1}^{N}{\left(x_{k}-\mu_{x}\right)}^{2}}\;\;\;\;\;\;\;RMS=\sqrt{\frac{1}{N}\;\cdot \;\sum_{k=1}^{N}{\left(x_{k}-\tilde{x}\right)}^{2}} \end{array}$$

where

- *N* is the total number of elements;
- $x_{k}$ is a generic element belonging to the set;
- $\mu_{x}$ is the mean value;
- $\tilde{x}$ is a reference value for the elements (i.e., in this case, the precise coordinates of the point).

In general, *STD* is considered a precision indicator, while *RMS* is an accuracy indicator. In this paper, we will not consider sample *STD*/*RMS* since we analyse data sets consisting of a single data survey for each period. Given *RMS* for direction *E* and *N*, we will also consider a single measure RMS2D, computed as follows:

(2) $$\begin{array}{c} RMS\;2D=2*\sqrt{{\left(RMS\;E\right)}^{2}+{\left(RMS\;N\right)}^{2}} \end{array}$$

All the processing described in the rest of this section was performed using the RTKLIB version 2.4.3 b34 by Tomoji Tamasu, Sakura, Japan, distributed under a BSD 2-clause license. In order to evaluate both accuracy and precision, the static tests were processed as kinematic ones, that is, by obtaining a point cloud as output, with the coordinates computed epoch by epoch. Mainly two types of processing are performed: a stand-alone positioning, which requires only the observable of the receiver under examination, and a relative post-processing, which involves not only the observables of the receiver under examination but also the ones of a base station. This second processing is also called post-processed kinematics (PPK). Unless otherwise specified, the options used for processing are the following: The ionosphere and troposhere models used are the Klobuchar [28] (the parameters of which are transmitted with the navigation message) and Saastamoinen [29], respectively. For every test, only the broadcast ephemeris were considered, and an elevation mask of 15° is also set.

Concerning the PPK elaborations, the solution type combined was set. In this modality, the observation data is processed through a Kalman filter in the forward direction, that is, starting with the beginning of the data and continuing through to the end. Backward mode is the opposite: data is run through the filter starting with the end of the data and continuing to the beginning. In combined mode, the filter is run both ways, and the two results are combined into a single solution.

### 2.4. Test 1: Results

The performance of the smartphone GNSS receiver in a kinematic contest can be analysed at two different levels: the pre-processing level and the positioning level. In both cases, to better understand its performance, the data coming from the smartphone (Xiaomi Mi 8) are compared with those produced by a Stonex S500 receiver, which is considered as a benchmark.

**Figure 3:**
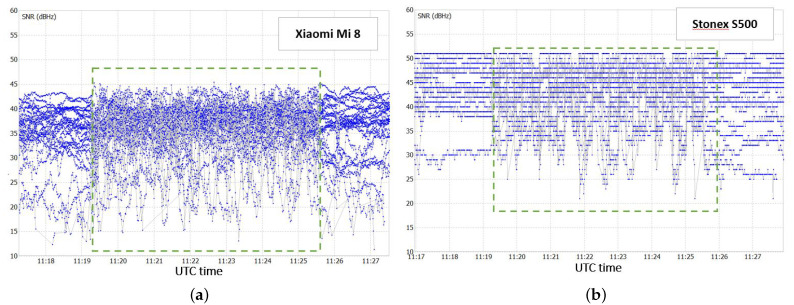
SNR for Xiaomi Mi 8 receiver (**a**) and for Stonex S500 receiver (**b**).

Concerning the pre-processing level, the quality of the incoming signal for the two receivers is compared. A good indicator of the incoming signal quality is the signal-to-noise ratio (SNR), which is a measure of the strength of the desired signal relative to background noise. SNR expressed in DB-Hz presents high values for a good incoming signal, and low values for a bad signal. The SNR values for the two receivers used in test 1 are shown in Figure 3.

Figure 3 shows that the Stonex S500 receiver has a much better SNR than the Xiaomi Mi 8 receiver. In fact, an average difference of about 10 DB-Hz between the two receivers is observed. From the SNR trend, it is also possible to recognise the static and kinematic parts of the survey. The kinematic part, highlighted in the green dashed box, actually presents a noisier SNR trend than the static parts located at the beginning and at the end of the test.

**Figure 4:**
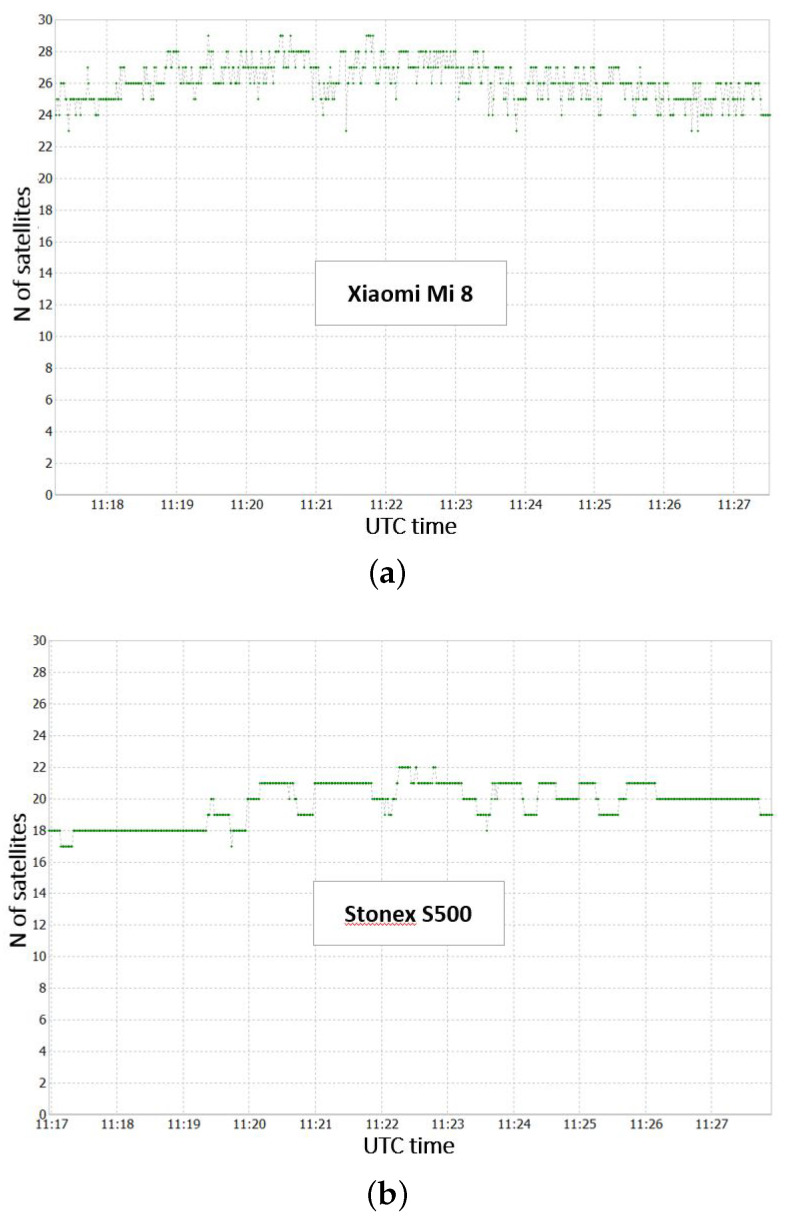
Number of satellite observed by Xiaomi Mi 8 receiver (**a**) and Stonex S500 receiver (**b**).

The number of satellites observed by the Xiaomi Mi 8 is greater than that of the Stonex S500 (see Figure 4), but much more variable over time. In fact, the Xiaomi Mi 8 receiver also acquires very noisy satellites (SNR < 25 DB-Hz) for short periods of time, which are ignored by the Stonex S500.

Concerning positioning, acquired data were post-processed with a common GNSS permanent station located about 200 m away from the test field, hereafter called LIGE [27]. The LIGE permanent station is equipped with the GNSS receiver u-blox ZED F9P coupled with the Hemisphere A45 antenna. For this case study, the solution type “forward” is set in order to simulate real-time conditions.

**Figure 5:**
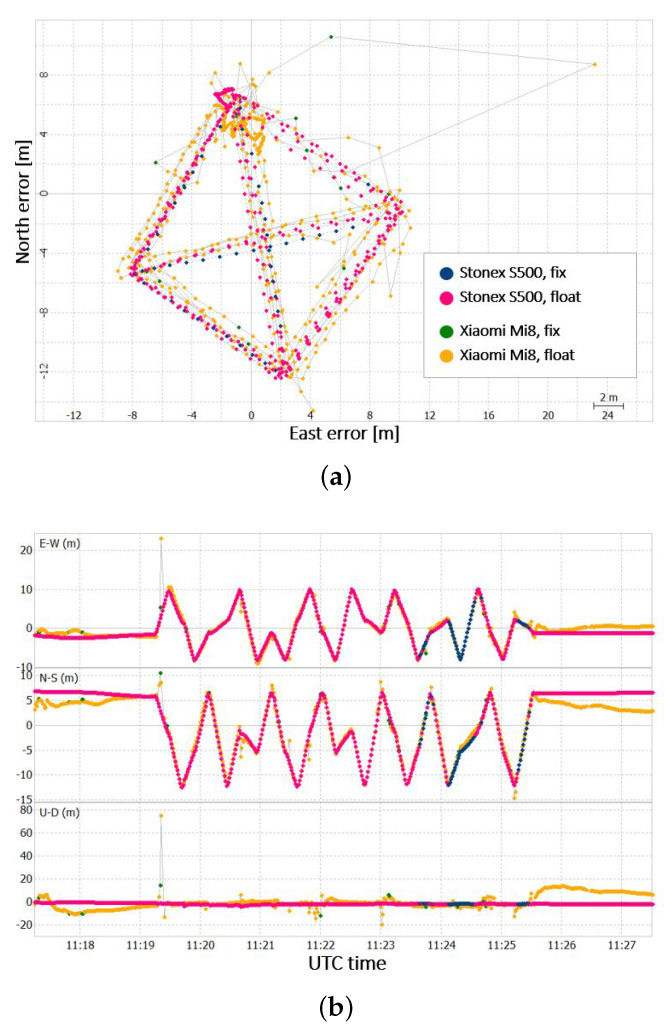
(**a**) Scatter plot of the positioning result for Xiaomi Mi8 and Stonex S500, and (**b**) time series of the positioning error for Xiaomi Mi8 and Stonex S500.

The positioning solutions obtained are shown in Figure 5.

The Stonex S500 receiver solution is more accurate, especially when considering the North and Altitude components. The two solutions can be also compared in terms of number of computed positions and percentage of fix and float solutions, as shown in Table 1.

The Xiaomi Mi 8 receiver presents fewer solutions than the Stonex S500. The poor quality of the GNSS measurement from the smartphone prevents the computation of the positioning for some epochs. Furthermore the Xiaomi Mi 8 solutions, coherently with other results obtained from the previous tests, present some outliers and false fixing solutions.

### 2.5. Test 2: Result

**Figure 6:**
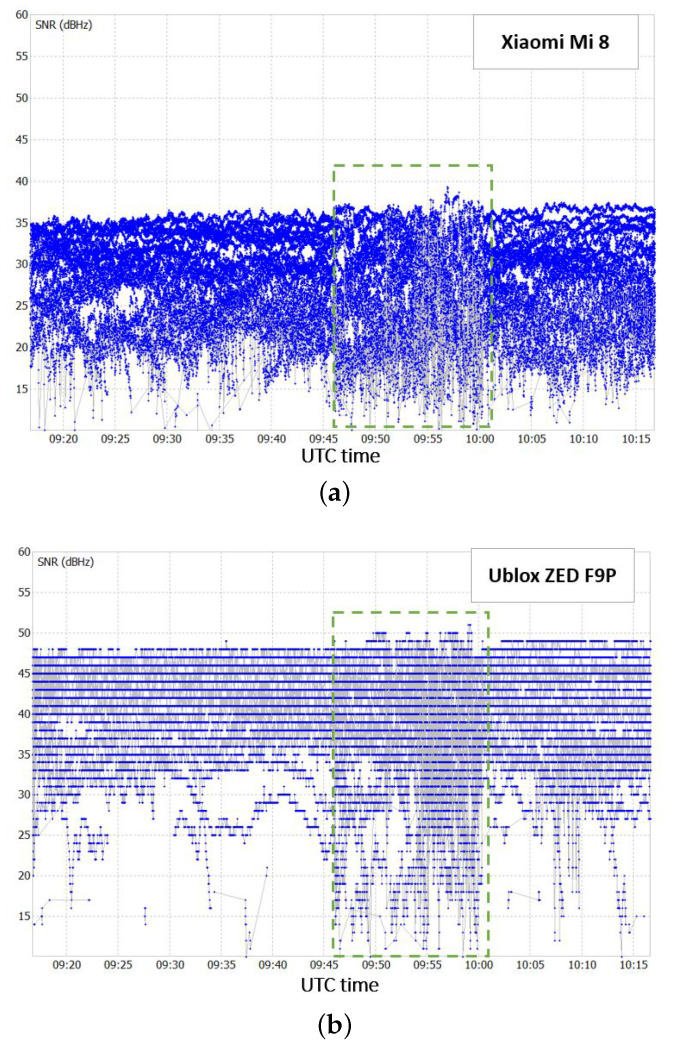
SNR for Xiaomi Mi 8 receiver and (**a**) for u−blox ZED F9P receiver (**b**).

Similarly to test 1, for this case, the performance of the Xiaomi Mi8 receiver is compared to that of the other device involved, the u−blox ZED F9P, at two different levels: the pre-processing and the positioning level. Once again, in the pre-processing level, the two receivers are compared in terms of the quality of the incoming signal. For this purpose, the SNR trend for the two receivers is shown in Figure 6.

The Xiaomi Mi 8 presents lower SNR values on average with respect to the u-blox ZED F9P. From the SNR trend of the two receivers, the period in which the multipath effect was induced with the metal plate is evident, that is, from 9:45 a.m. to 10:00 a.m. UTC (green dashed boxes in Figure 6). In this time interval, as expected, the signal acquired is degraded. This results in a general lowering of SNR values, and a significantly noisier trend.

**Figure 7:**
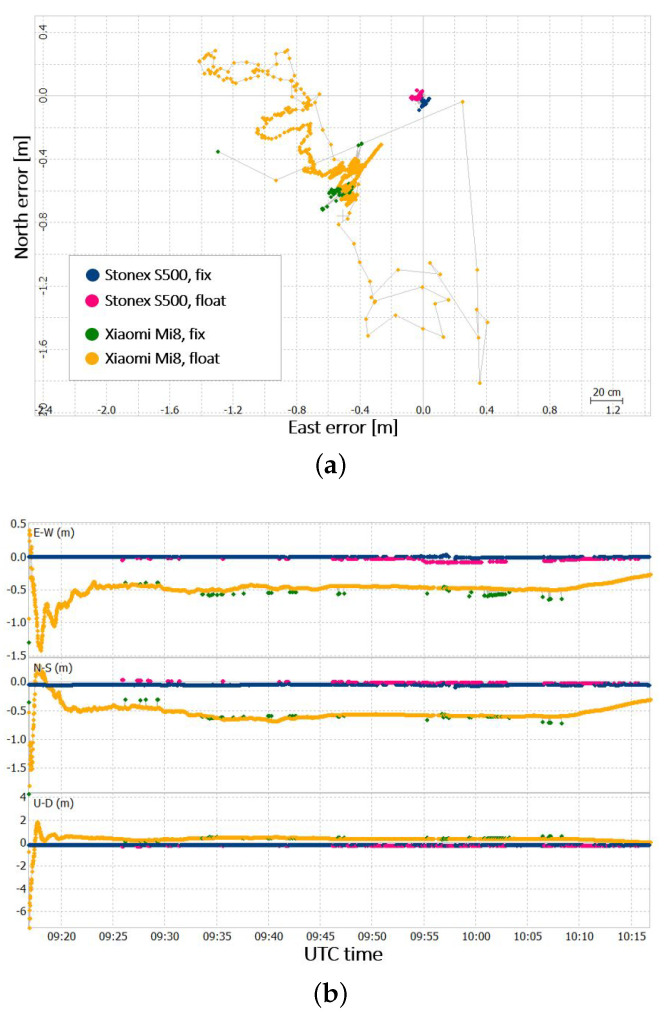
(**a**) Scatter plot of the positioning error for Xiaomi Mi8 and u−blox ZED F9P; (**b**) time series of the positioning error for Xiaomi Mi8 and u−blox ZED F9P.

Concerning the positioning part, the data were again post-processed with the LIGE permanent station. Similarly to test 1, for this case study the solution type chosen is also forward. Figure 7 shows the obtained positioning results in terms of errors with respect to the precise coordinates of the point.

The statistics values, in terms of RMS and standard deviation with respect to the precise coordinates of the receivers, are reported in Table 2.

In this case study, the solution for the Xiaomi Mi8 receiver has a convergence time of about 5 min. After this time interval, the standard deviations of the solution are 0.051 m, 0.08 m, and 0.093 m for the East, North, and Height components, respectively. The solutions therefore present a centimetre-level accuracy; nevertheless, the u-blox ZED F9P solution results were more accurate. After the convergence time, the Xiaomi Mi8 solution has average deviations from the precise point coordinates of −0.460 m, −0.549 m, and 0.390 m for the East, North, and Height components, respectively. It can be stated that the solution presents a decimetre precision level, but once again, the u-blox ZED F9P solution results are more precise.

Consistently with the results already obtained, for the other case studies, the Xiaomi Mi8 has a lower number of solutions (3473 against 3501) and a lower percentage of fixed solutions (2.4% against 81.3%).

For this case study, it is also interesting to analyse the solution in the time interval in which the multipath was induced (i.e., between the 9:45 and the 10:00 UTC). For the u-blox ZED F9P, the solution results are particularly degraded (see Figure 7b), and almost the totality of the float solutions obtained for this receiver are in this time interval. Concerning the Xiaomi receiver, the solution does not seem particularly degraded in this time interval with respect to the entire test period; nevertheless, in this interval, some false fixed solutions can be observed.

## 3. The GNSS RTK IoT System

As discussed in Section 2, GNSS positioning with smartphones suffers from false fixed solutions, and outliers that compromise the robustness of the positioning itself. Those false fixed solutions can be caused by many factors, including the poor quality of the GNSS observables and the presence of external interferences such as the multipath effect. In this section, we present a solution to increase the robustness of GNSS RTK positioning. The architecture of the GNSS RTK positioning procedure that we used to process GNSS data coming from Android smartphones consists of the following components:

- A smartphone for data acquisition (the rover);
- A GNSS base station;
- A remote server for RTK processing.

**Figure 8:**
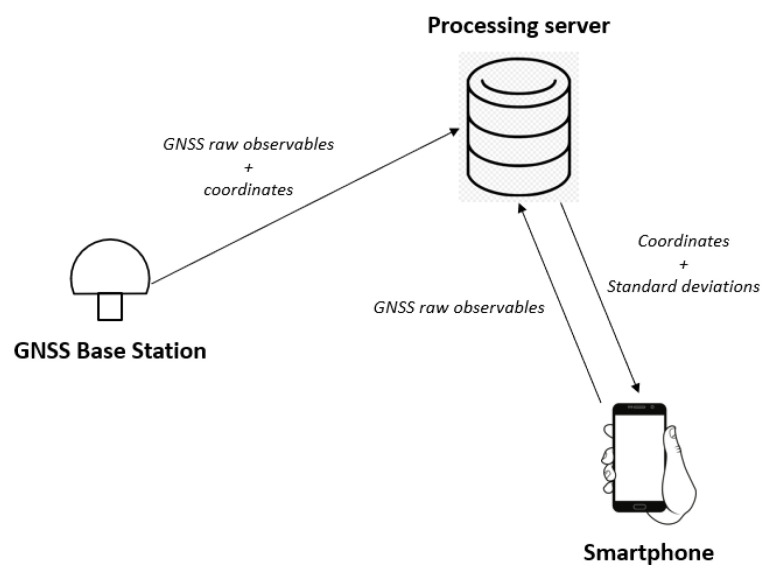
Proposed architecture for robust RTK positioning with smartphone.

The smartphone is supposed to be equipped with an Android app capable of reading GNSS raw observables, packing them in a proper way, and sending them to a server running RTKLIB through a TCP socket. The base station must be capable of sending, in real time, its observables together with its precise coordinates to the server. The server is supposed to be capable of reading the input streams from the smartphone and from the base station, processing them with RTKLIB, and then returning the coordinates to the smartphone. The resulting architecture is shown in Figure 8.

The proposed system works with an ad-hoc GNSS base station assembled with a u-blox ZED F9 receiver, but it can be generalised by using an NRTK network instead.

The central component of the architecture, as shown in Figure 8, is a remote server for GNSS positioning. The server runs the RTKLIB library for data processing and communicates in real time with both the smartphone and the base station though TCP/IP sockets. The RTKLIB version installed in the server was properly modified in order to make it work with GNSS data coming from a smartphone in real time. This new version of RTKLIB not only can process data coming from a smartphone in real time but also implements an algorithm for detecting and mitigating the multipath effect. This algorithm, which is the main contribution of this work, is explained in depth in the following section.

## 4. Multipath Mitigation Algorithm

One of the major external interferences that degrade GNSS positioning is multipath. The effect of this interference is well described by its name: a satellite-emitted signal arrives at the receiver from different directions (i.e., following different paths). The multipath effect is mainly caused by reflecting surfaces near the receiver. For this reason, it frequently happens in urban canyons, a typical scenario in which smartphones are used. The multipath effect may also occur when using low-cost GNSS receivers equipped with low-quality antennas, such as the smartphone’s. For this reason, a good strategy to improve the robustness in GNSS positioning from Android devices should aim at mitigating the multipath error.

Multipath mitigation can be performed at the antenna, receiver (signal processing), and navigation solution level [30].

In this work, multipath mitigation is approached at the software level, using an algorithm, called MDP (multipath detection parameter) conceived by Gter. In the following section, the algorithm is explained in detail, together with its implementation in RTKLIB.

### 4.1. The MDP Algorithm

The MDP algorithm consists of two main parts: the first one concerning multipath detection, and the second one concerning multipath mitigation. The multipath detection part is based on the SNR (signal-to-noise ratio) value and a new variable called MDP (multipath detection parameter). The aim of the MDP value is to create a variable representative for the multipath effect for single-frequency GNSS receivers, starting from the observation equation. The observation equations for GNSS are as follows:

(3) $$\begin{array}{c} P\left(t_{1}\right)=\rho \left(t_{1}\right)+\Delta T_{r}^{s}\left(t_{1}\right)+Ion\left(t_{1}\right)+Trop\left(t_{1}\right)+Mult\left(t_{1}\right)+\epsilon_{1}\\ L\left(t_{1}\right)=\rho \left(t_{1}\right)+\Delta T_{r}^{s}\left(t_{1}\right)-Ion\left(t_{1}\right)+Trop\left(t_{1}\right)+mult\left(t_{1}\right)+\lambda A+\epsilon_{2} \end{array}$$

where

- $P(t_{1})$ is the pseudorange observable at instant $t_{1}$;
- $L(t_{1})$ is the carrier phase observable at instant $t_{1}$;
- $\rho (t_{1})$ is the geometric range between the satellite and the receiver at instant $t_{1}$;
- $\Delta T_{r}^{s}\left(t_{1}\right)$ is the global time unknown at instant $t_{1}$;
- $Ion(t_{1})$ is the ionospheric effect on the signal at instant $t_{1}$;
- $Trop(t_{1})$ is the tropospheric effect on the signal at instant $t_{1}$;
- λA is the phase ambiguity;
- $Mult(t_{1})$ is the code multipath;
- $mult(t_{1})$ is the phase multipath;
- $\epsilon_{1}$ and $\epsilon_{2}$ represent residual errors due to noise.

One common approach to isolate the multipath effect, from Equation (3), is to compute the difference [31]. The result is the so-called sentinel variable:

(4) $$S\left(t_{1}\right)=P\left(t_{1}\right)-L\left(t_{1}\right)=2Ion\left(t_{1}\right)-\lambda N+\left(Mult\left(t_{1}\right)-mult\left(t_{1}\right)\right)+\epsilon$$

where ϵ represents residual error. The definition of MDP variable requires two assumptions. Considering a sufficiently high acquisition rate (i.e., 1 Hz), we can assume that:

- The ionosphere effect is equal between two consecutive epochs;
- The phase ambiguity is equal between two consecutive epochs.

Based on these assumptions, the MDP variable is defined as follows:

(5) $$MDP=S\left(t_{2}\right)-S\left(t_{1}\right)=\left\{\left[Mult\left(t_{2}\right)-mult\left(t_{2}\right)\right]-\left[Mult\left(t_{1}\right)-mult\left(t_{1}\right)\right]\right\}+\epsilon^{\prime }$$

where $\epsilon^{\prime }$ represents the difference of residual errors. Hence, as shown in Equation (5), the MDP variable is representative of the effect of the multipath effect and residual errors due to noise.

### 4.2. MDP: Detection Algorithm

The detection algorithm compares the SNR and MDP parameters to static and dynamic threshold values as explained below.

#### 4.2.1. SNR Threshold

The multipath detection part proposed in the MDP algorithm also exploits the SNR value, which is not a specific indicator for multipath but for noise in general. The aim of checking the SNR mask as well is to identify outliers that can be caused by multipath or other external interference or can refer to corrupted data. More specifically, for the SNR parameter, a static threshold is set before running the positioning procedure. The value to be assigned to the SNR threshold depends on several factors, such as the quality of the GNSS antenna and the environmental conditions of the survey. The optimal value to assign to the threshold is still an object of research. In this work, it is determined experimentally by trying different configurations, as explained in Section 5. Epoch by epoch, for each acquired observable, the SNR value is compared to the chosen threshold: if the SNR value is lower than the threshold, the observable is flagged with a so-called SNR flag.

#### 4.2.2. MDP Threshold

The MDP is a parameter specifically designed to identify data affected by multipath. Under normal conditions, (i.e., absence of multipath), the variable MDP (measured in metres) is expected to have a white noise trend, and its values depend on the entity of residual errors due to noise. In case of incoming multipath, the MDP variable is expected to have some outliers. The aim of the MDP threshold is to identify those outliers, as they indicate multipath-affected data. Two different MDP thresholds were introduced in this work: a static one and a dynamic one. The static MDP threshold is used in a similar way to the SNR threshold. The optimal MDP threshold value is still an object of research, and in this work, it is determined experimentally by trying different configurations, as discussed in Section 5. Once the MDP static threshold is set, epoch by epoch, for each acquired observable, the MDP value is computed and compared to the threshold. The observable is then flagged with a so-called MDP flag if:

(6) |MDP|≥mdpthreshold

Concerning the MDP, an adaptive threshold was also proposed. This adaptive threshold is computed considering a statistical analysis on previous epochs. The heuristic is based on an additional parameter, *N*, that defines the length of the observation window (number of epochs to be analysed). As the aim of the MDP threshold is to detect outliers, Chebyshev’s inequality is used for its definition. In fact, Chebyshev’s inequality states that, considering a broad range of probability distributions, 88.89% of values lies within three standard deviations of the mean [32]. The MDP adaptive threshold is then defined as follows:

(7) mdpthreshold=μ±3σ

where

- μ is the mean MDP value on *N* previous epochs;
- σ is the standard deviation of the *N* previous MDP values.

In this case, the observable is flagged if:

(8) MDP≤μ−3σ∨MDP≥μ+3σ

#### 4.2.3. Initialisation Phase

The detection part of the algorithm, when the MDP dynamic threshold is chosen, requires an initialisation time equal to *N* times the acquisition rate. Therefore, for example, if the acquisition rate is 1 Hz and *N* is equal to 60, the detection algorithm needs 1 min to reach a complete operating status.

#### 4.2.4. Detection Phase

Based on the current values of the MDP and SNR flags, the detection algorithm implements three different criteria to state if the observable is affected by multipath. The considered criteria are the following:

- Criterion 1: Only MDP flag;
- Criterion 2: MDP flag and SNR flag.

For each epoch, using this procedure, a set of observables potentially affected by multipath is identified. The detection phase is used then to trigger the mitigation procedure.

### 4.3. MDP: Mitigation Algorithm

The aim of the mitigation algorithm is to retrieve a more accurate and precise GNSS positioning, taking into account multipath-affected observables. One possible way to mitigate the multipath effect is to exclude the multipath-affected observables. This heuristic runs the risk of excluding too many GNSS observables, hence producing a poor-quality result due to missing redundancies in the observables. In order to avoid this scenario, a good way to proceed is to consider different associated weights to the multipath-affected GNSS observables.

Since the MDP is integrated in RTKLIB, the new proposed weight to be associated with the multipath-affected observables is based on the weight that the software associates with the observables. As previously described, RTKLIB uses a weight matrix defined as follows:

(9) $$W=diag\left(\sigma_{1}^{-2},\sigma_{2}^{-2},...,\sigma_{n}^{-2}\right)$$

In Equation (9), $\sigma_{n}^{2}$ is the variance associated to the *n* observable defined as:

(10) $$\sigma_{meas}^{2}=F^{s}R_{r}\left(a_{\sigma }^{2}+\frac{b_{\sigma }^{2}}{sinEL_{r}^{s}}\right)$$

where

- $F^{s}$ is the satellite system error factor, which is equal to 1 for GPS, Galileo, QZSS; equal to 1.5 for BeiDou; and equal to 3 for GLONASS;
- $R_{r}$ is the code/carrier-phase error ratio;
- $a_{\sigma },b_{\sigma }$ are the carrier-phase error factors a and b in metres.

Furthermore, the software adds to this variance other contributions, so the final equation becomes:

(11) $$\sigma_{obs}^{2}=\sigma_{meas}^{2}+\sigma_{eph}^{2}+\sigma_{ion}^{2}+\sigma_{trop}^{2}+\sigma_{bias}^{2}$$

where

- $\sigma_{eph}$ is the standard deviation of ephemeris and clock error in metres;
- $\sigma_{ion}$ is the standard deviation of ionosphere correction model error in metres;
- $\sigma_{trop}$ is the standard deviation of troposphere correction model error in metres;
- $\sigma_{bias}$ is the standard deviation of code bias error in metres.

In order to take into account the multipath effect, the variance of the observables identified as potentially affected by multipath is incremented, adding a new component. This component is based on the sigma-ϵ model [33,34] used to weight GNSS observables by means of their SNR value. A new term has been added to this model in order to also take into account the multipath effect by means of the MDP value. The resulting term, called MDP variance, is expressed by:

(12) $$\sigma_{mdp}^{2}=mdp^{2}+C\;\cdot \;10^{-\frac{SNR}{10}}$$

where *C* is a model parameter equal to 0.244 m^2^ DB-Hz for the L1 frequency. Formula (11) is then revised as follows:

(13) $$\sigma_{obs}^{2}=\sigma_{meas}^{2}+\sigma_{eph}^{2}+\sigma_{ion}^{2}+\sigma_{trop}^{2}+\sigma_{bias}^{2}+\sigma_{mdp}^{2}$$

The proposed weight model is defined in such a way as to amplify the impact of the multipath effect (MDP variable) with respect to external noise (SNR). Since the weight associated to the observables is equal to the inverse of its variance (see Equation (9)), the weight of the observable decreases as its MDP value increase. Furthermore, using the proposed MDP variance, the multipath-affected observables are weighted in a different way depending on their MDP values: observables affected by a large multipath error (high MDP value) will have a lower weight with respect to observables with a lower multipath error (i.e., lower MDP value). Furthermore, the MDP variance takes into account the SNR value as well. As the SNR value decreases (i.e., the observable noise increases), the MDP variance associated to the variable increases, and consequently, its weight decreases. An experimental validation of the proposed algorithm will be presented in the next section.

## 5. Experimental Results

In this section, the effects on GNSS positioning by smartphones introduced by the application of the MDP algorithm are discussed. In particular, the performance of the algorithm, in terms of multipath detection and mitigation, under varying configuration parameters is detailed. For this purpose, both static and kinematic data sets are analysed. The selected case studies are used to test the main features of the proposed architecture. They are both processed using the LIGE base station, developed for this work. They require the use of our Android app installed on a device and the modified RTKLIB version for data processing. Although the proposed architecture has been designed for real-time applications, in order to evaluate the performance of the MDP algorithm, several post-processing elaborations were computed, all having the same input data and therefore being under the same contextual conditions.

The MDP algorithm present several input parameters to tune the computation phase. First of all, in the detection step, one out of three criteria must be selected. Moreover, the strategy for the MDP threshold computation must be chosen between the static and the adaptive. The value chosen as static threshold remains constant for the entire computation. For the adaptive threshold value, the number of previous epochs should be set. The MDP threshold will then depend on a statistical analysis computed on the previous MDP values following Equation (8). Finally, the SNR threshold must be set.

The values to be associated with the various parameters to achieve optimal results may depend on various factors (e.g., environmental conditions and hardware characteristics of the receiver) and are still an object of research. For the two case studies, some PPK processing was performed considering different combinations of the parameters, as explained later. Concerning the rest of the processing options used in RTKLIB for PPK positioning, the ionosphere and troposhere models used are the Klobuchar and Saastamoinen, respectively. Only the broadcast ephemeris were considered, and an elevation mask of 15° is also set. The solution type selected is “forward” for both case studies.

### 5.1. Static Test

**Figure 9:**
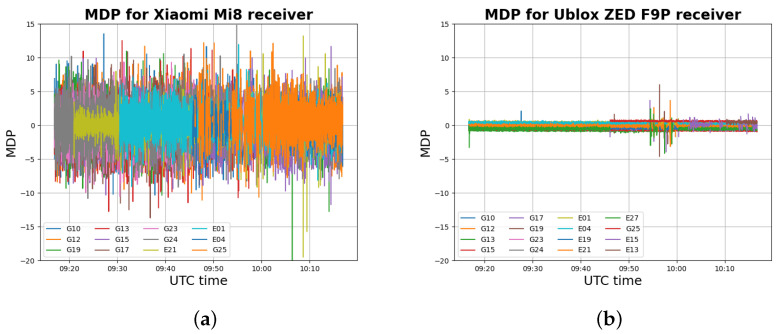
MDP values for Xiaomi Mi8 receiver (**a**) and for Ublox ZED F9P receiver (**b**) for the static data set.

The static data set refers to the acquisition described in Section 2. The MDP trend is presented for both the receivers used, with the purpose of understanding the MDP performance in detecting multipath errors for smartphone receivers with respect to a classic GNSS receiver. Figure 9 shows the MDP values detected by the Xiaomi Mi8 and the u-blox ZED F9P.

The MDP variable is representative of the multipath effect and residual errors due to external noise (see Equation (5)). Figure 9 shows that the MDP variable for the u-blox ZED F9P receiver has some peaks between the 9:45 and 10:00 UTC, that is, when the multipath effect was induced, as discussed in Section 2. The MDP variable can then be considered a reasonable multipath indicator for this kind of receiver. For the Xiaomi Mi8 receiver, the MDP variable presents a noisy trend with higher values in modulus. Differently from the u-blox ZED F9P, the Xiaomi Mi8 MDP trend has more diffuse peaks and is not particularly concentrated between 9:45 and 10:00 UTC. Nevertheless, a couple of peaks can be noted for the Xiaomi Mi8 in that interval. The noisier trend of the Xiaomi Mi8 MDP variable with respect to the u-blox ZED F9P can be explained by higher values of the external noise caused by the poor quality of the smartphone’s antenna. This makes the MDP variable less effective in multipath identification for smartphones receivers.

For evaluating the performance of the MDP algorithm for the smartphone receiver, processing was computed under changing configuration parameters. Data were processed using all three criteria proposed for multipath detection. For every criterion, both static and adaptive MDP thresholds were used, as specified below.

- Concerning the static threshold, six values were selected on the basis of the MDP trend (Figure 9a). The chosen values of the MDP static threshold are 2.5, 3.0, 3.5, 4.0, 4.5, and 5 m. These values have been selected after several tests, as discussed in [27], and indications from the literature.
- For the adaptive threshold, six different windows (number of epochs) are selected: 10, 20, 30, 40, 50, and 60. The window size times the acquisition rate determines an initialisation time in which the algorithm cannot be applied. Considering that all smartphones have an acquisition rate of 1 Hz, an initialisation time no longer than 1 min (i.e., 60 epochs) was considered in the experiments.
- Finally, considering the SNR trend for the Xiaomi Mi8 receiver (Figure 6a), four values of the SNR threshold are considered: 20, 25, 30, and 35 DB-Hz. Again, these values have been selected after several tests, as discussed in [27], and indications from the literature.

Combining all these option values, we considered 108 different experimental tests. Hereafter, the most interesting results are reported. The interested reader may refer to Lorenzo Benvenuto’s PhD thesis for a complete description of the experiments [27].

**Figure 10:**
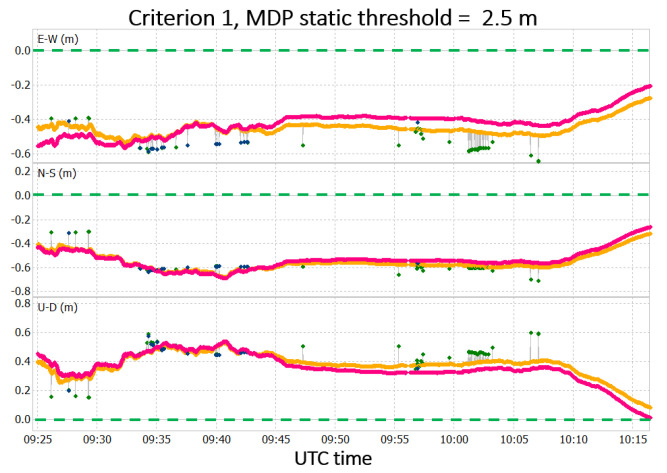
Results obtained after MDP algorithm application using criterion 1 and static MDP threshold equal to 2.5 m.

Considering criterion 1, the solutions obtained for the static MDP threshold values, compared with the solution without the MDP algorithm application, are shown in Figure 10.

In Figure 10, the solutions obtained after the MDP algorithm is applied are depicted in pink and blue for float and fixed solutions, respectively, while the solution obtained without the application of MDP algorithm is depicted in yellow and green for float and fixed solutions, respectively. In the figure, the precise coordinates of the point are also highlighted with the dashed green line. The data set presents a convergence time of about 5 min, which is not considered for the results analysis.

The solution, after the application of the MDP algorithm, has slight improvements in terms of accuracy, especially in the case of MDP threshold equal to 2.5. Considering the planimetric accuracy, an improvement of 5 cm is obtained. Over all the threshold values considered for this criterion, this is the best result reached. The worst result obtained is the one with an MDP threshold value of 5.0 m (see [27]).

**Figure 11:**
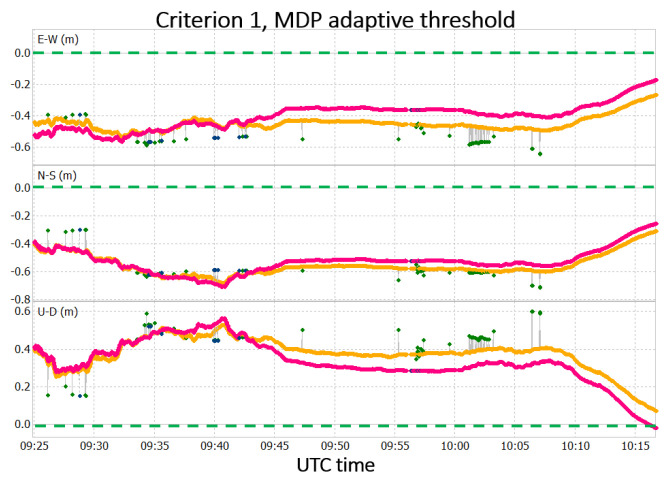
Results obtained after the MDP algorithm application using criterion 1 and adaptive MDP threshold.

As additional experiment, the data set has been processed considering the adaptive MDP threshold with a time window containing 30 epochs. This window size has been selected after additional experiments (from 10 to 60 epochs) discussed in [27]. The positioning results concerning the processing executed with criterion 1 and the adaptive MDP threshold compared with the solution without the MDP algorithm application are reported in Figure 11.

The solution obtained with the application of the MDP algorithm is in pink and blue, and the solution obtained without its application is in yellow and green; the precise coordinates of the point are represented by the dashed green line. In this case, improvements are present in the solution accuracy not only with respect to the case without the MDP algorithm application, but also with respect to the case with the static MDP threshold equal to 2.5 m. The difference in terms of RMS for the three cases is reported in Table 3.

The results shown in Table 3 can be explained by the fact that the adaptive threshold is more selective than the static threshold in the identification of data potentially affected by multipath. When dealing with very noisy GNSS observables, such as those produced by smartphones, the usage of a static MDP threshold may lead to consideration of any data that are not affected by multipath as outliers in the MDP trend. Reducing the weights of those data still leads to some benefits in terms of positioning accuracy, but those benefits are lower with respect to the ones obtained if only multipath-affected observables (i.e., outliers in the MDP trend) will be assigned a reduced weight. Concerning the precision of the solutions, appreciable differences are noted in the STD of the solutions, so it can be stated that the MDP algorithm does not lead to improvement in positioning precision.

**Figure 12:**
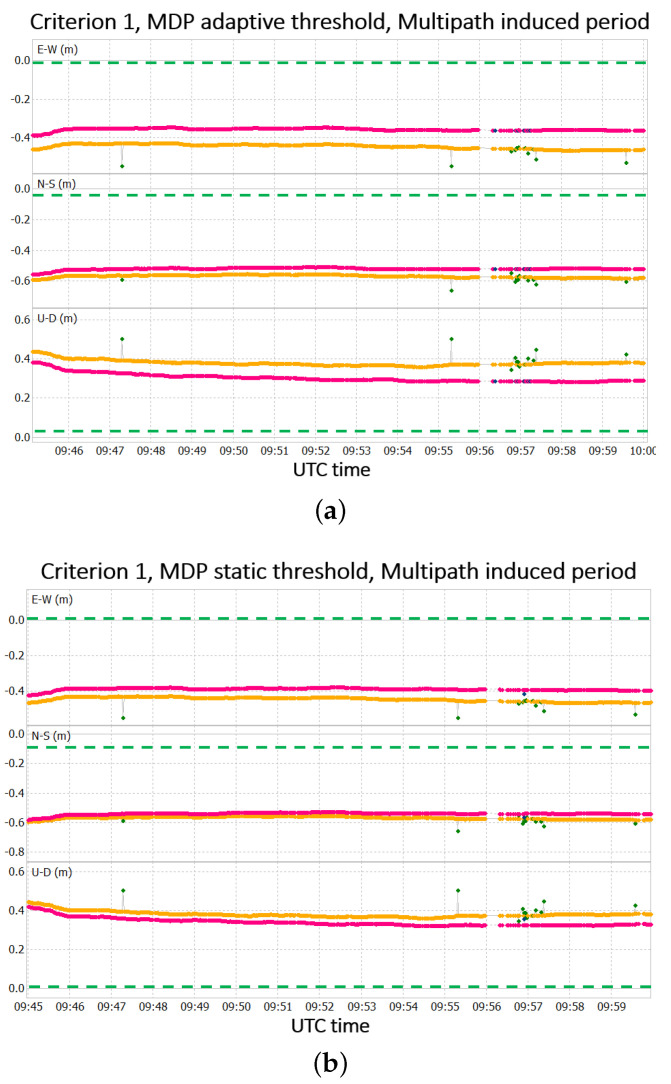
Results obtained after application of the MDP algorithm using criterion 1 with an adaptive MDP threshold (**a**) and static MDP threshold (**b**), referring to the multipath-induced interval.

From Figure 10 and Figure 11, it can be seen that the best improvements are obtained during the period of the induced multipath (i.e., from 9:45 to 10:00 UTC). The solution obtained for both adaptive and static threshold in that time interval are depicted in Figure 12. For the rest of the period, the solutions with and without the application of the MDP algorithm are quite similar.

In Figure 12, the solution obtained with the MDP algorithm presents an unstable set of fixed solutions. However, fixed solutions obtained without the MDP algorithm often turned out to be false solutions. Indeed, their RMS is in the order of tens of centimetres, while, for fixed solutions, the expected RMS should be in the order of few centimetres. In this sense, an unstable set of results that is still consistent with the rest of the obtained positions seems to be preferable to false fixed solutions. Therefore, the MDP heuristics seems to increase the robustness of the positioning process in the selected test battery.

The differences in terms of RMS for the adaptive threshold case and static one are reported in Table 4.

Similarly to the whole test period, in this time interval, the solution accuracy particularly increases when the adaptive threshold is considered. Considering that the multipath error was manually induced in this time interval, the MDP algorithm seems to be effective in mitigating this type of error.

In criterion 2, the observable is considered to be affected by multipath if its SNR value is lower than its threshold, and at the same time, the MDP value in modulus is higher than the current MDP threshold. Considering the static MDP threshold, the major improvements are obtained for MDP threshold equal to 2.5 m, so the impact of the SNR value is discussed only for this situation. The statistics in terms of the RMS referred to in our experiments are reported in Table 5.

In the case of the SNR threshold set to 20 DB-Hz, very low improvements can be noted in term of accuracy with respect to the solution without the MDP algorithm. This means that very few observations have the condition on MDP and SNR simultaneosly. Compared to the results in Table 3, the introduction of a very low SNR threshold worsens the performance of the algorithm in improving the accuracy of the solution. In addition, no significant improvements in terms of solution robustness can be observed in this case. Considering the case with SNR threshold equal to 35 DB-Hz instead, some improvements in terms of accuracy can be observed. Furthermore, if an SNR threshold equal to 35 DB-Hz is considered, the number of false fixed solutions is reduced with respect to the “no MDP” case. This means that the solution robustness is increased.

Some additional tests for criterion 2 considered the adaptive MDP threshold. In these cases, no significant differences were obtained with respect to the results for criterion 1. Indeed, as previously noted, the MDP adaptive threshold, differently from the static one, only selects a few outliers in the MDP trend. The SNR threshold instead selects many pieces of data, especially if its value is set to 35 DB-Hz. Considering that for criterion 2, the MDP and SNR condition must be verified at the same time, it is reasonable to deduce that the discarded data in this case are the same as those selected via criterion 1 where the SNR is not checked for the detection part of the algorithm.

Summarizing, it is possible to state that, considering criterion 2, the usage of the SNR values for the detection phase of the MDP algorithm has advantages for the mitigation performance of the algorithm itself, if a static MDP threshold is considered. Those benefits regard the position accuracy and robustness, and they are more evident for increasing values for the SNR threshold. No differences are introduced by the usage of SNR in the detection part if the MDP adaptive threshold is selected. No appreciable differences can be observed, for all the processing executed for this criterion, in terms of STD.

### 5.2. Kinematic Test

The kinematic data set refers to the pedestrian acquisition described in Section 2. As for the static case study, the MDP trend is presented for both receivers, with the purpose of understanding the MDP performance of detecting multipath for smartphone receivers compared to more traditional GNSS receivers.

**Figure 13:**
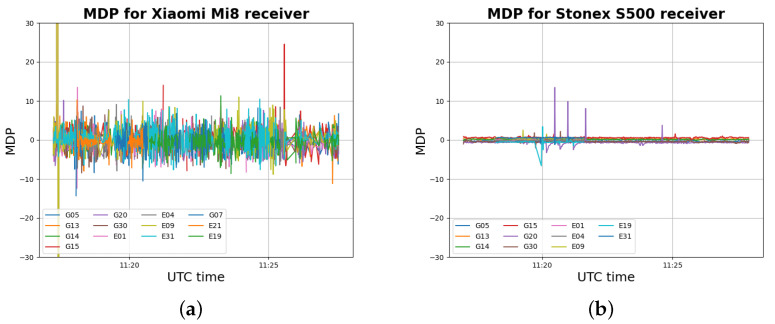
MDP values for Xiaomi Mi8 receiver (**a**) and for Ublox ZED F9P receiver (**b**) for a kinematic data set.

Similarly to what observed for the static case study, the MDP variable for the smartphone receiver (Figure 13a) is noisier compared to of a classic GNSS receiver. The chosen values for the MDP static threshold are: 2.5, 3.0, 3.5, 4.0, 4.5, and 5 m. For the adaptive threshold, considering the results obtained for the static case study, only 30 previous epochs were considered for the threshold computation. Finally, considering the SNR trend for the Xiaomi Mi8 receiver (Figure 3a), four values of the SNR threshold were considered: 20, 25, 30, and 35 DB-Hz. Combining all these option values, 63 different processing results were obtained. Hereafter, the obtained results are reported.

The best results for criterion 1 were obtained considering the MDP threshold equal to 2.5 m. The RMS results for the static part are reported in Table 6. In order to justify the better improvements obtained with the MDP threshold equal to 2.5 m with respect to the other tested values, the worst result obtained (i.e., MDP threshold equal to 5.0 m) is also reported in the table.

As shown in Table 6, some improvements in terms of accuracy are obtained after the MDP algorithm application. The MDP algorithm seems particularly effective in improving the accuracy of the solution when the MDP threshold equal to 2.5 m is considered.

Concerning the kinematic part, the application of the MDP algorithm produces some local benefits in the solution with the elimination of some outliers observed in the “NO MDP” solution. This means that in those intervals, the robustness of the solution is increased. Nevertheless, the MDP algorithm seems to introduce some outliers that were not observed in the “NO MDP” solution due to the fact that some data not affected by multipath were under-weighted by the algorithm. Using the static MDP threshold for multipath identification seems to work well if the receiver is static, but it seems to be not so effective if the receiver is moving.

Criterion 1 coupled with the adaptive MDP threshold was also tested for this case study. Unlike the results obtained for the static MDP threshold, the solution accuracy in this case is degraded if the static part of the data set is considered, as shown in Table 7.

This result highlights that for this case study, the adaptive SNR threshold seems to be less effective in multipath recognition. Nevertheless, a significant improvement in the position accuracy can be noted for the East and North components after 11:18 UTC. After this instant, the planimetric accuracy is improved by about 11 cm, as the 2D RMS passes from 3.642 m to 3.528 m. This means that the bad performance of the MDP multipath detection is limited to the first few epochs of the data set.

Concerning the kinematic part of the data set, a similar behaviour to the one observed for the static MDP threshold is obtained. Nevertheless, in this case, some improvements are shown. The adaptive MDP threshold solution does not in fact have some of the outliers that the static MDP solution presents, especially for the Height component. This means that if the criterion 1 is considered for kinematic data sets, the usage of the adaptive MDP threshold seems preferable to the usage of a static MDP threshold [27].

Hereafter, the results for criterion 2 are exposed with particular attention to the role played by the SNR threshold in the detection part of the MDP algorithm. Among all the MDP static threshold values considered, the best results were obtained for the threshold value equal to 2.5 m, which is consistent with the results observed for criterion 1. Varying the SNR threshold value, it was noted that the solution accuracy, at least for the static part, improves as the threshold value increases. Further processing was carried out by raising the SNR threshold value from 35 to 40 DB-Hz, but no significant differences were noted, as shown in Table 8.

From the SNR value reported, we observe that the accuracy of the solution in this interval is improved by about 19 cm, 35 cm, and 52 cm for the East, North, and Height components, respectively. The planimetric accuracy of the solution is improved by 73 cm. It is also interesting to note the difference in RMS between this solution and the one obtained for criterion 1 (for comparison, see Table 6), that is, without the usage of SNR for the multipath detection part. Concerning the kinematic part, no substantial differences are observed with respect to the criterion 1 case. It can be stated then that the solution obtained for criterion 2 is better in terms of accuracy compared to the one obtained for criterion 1, in the case of an MDP static threshold, especially for the static part of the test. Therefore, in static condition, considering both the SNR and MDP values for the detection part of the MDP algorithm increases the performance of the algorithm itself. The situation is different for the kinematic part, in which no differences are noted. In this case, it seems that using the SNR value does not provide any advantage to the detection part of the MDP algorithm.

The test with criterion 2 also involved the usage of the adaptive MDP threshold. In this cases, varying the SNR threshold value, no significant differences were reported with respect to the results already obtained for criterion 1, so the results are not shown. It can then be stated that for criterion 2, if the adaptive MDP threshold is considered, the usage of the SNR value does not improve the performance of the detection part of the MDP algorithm.

The kinematic part of this data set shows behaviour analogous to that obtained for the processing carried out for criteria 1 and 2. No further improvements are observed for this case study, and the same considerations, already discussed for the other cases, are valid also for this obtained result.

## 6. Conclusions and Perspectives

The obtained results are very encouraging and promising. In particular, they confirm that the proposed solution is capable of increasing both accuracy and robustness in RTK positioning from Android devices. Nevertheless, the validation process of this solution shall continue applying the MDP algorithm in several other tests, considering a wider range of boundary conditions.

The MDP algorithm detection capabilities need to be studied in deeper detail, especially for Android GNSS receivers that present very noisy observables. Regarding this topic, in this work, both a static and adaptive MDP threshold were tested. Concerning the static MDP threshold, it was observed that the performance of the algorithm improves as the threshold value decreases. The optimal threshold value found for both the data sets is 2.5 m. Regarding the multipath effect identified by outliers in the MDP trend (Figure 9a and Figure 13a), and also considering the high noise of the smartphone’s GNSS observables (which makes the MDP variable higher in modulus), the observables with MDP values lower than 2.5 m cannot be considered affected by multipath. The adaptive MDP threshold was also tested, showing interesting results. In this case, each observable has its own threshold value, and multipath identification seems to be more effective. Lastly, the effects of SNR were tested for the detection part of the MDP algorithm combining SNR and MDP thresholds by means of a different strategy, that is, criterion 2. With criterion 2, the usage of SNR provides benefits to the detection capabilities of the algorithm. The solution accuracy and robustness are increased, especially when high values of SNR threshold (i.e., 35 DB-Hz) are considered. A less interesting criterion, with MDP or SNR, has been considered in [27].

Among the future developments of this work, it is worth mentioning a deeper investigation of the adaptive version of the MDP algorithm and, in particular, dealing with epochs with several missing observations for some specific satellites.

Another future development of this work is related to GNSS and INS (inertial navigation system) integration using data from the inertial sensor embedded in the smartphone. Integrating the positioning derived from the application of the MDP algorithm with inertial data through a loosely coupled approach should further increase the robustness of the resulting solution.

## Figures and Tables

**Table 1:** Test 1: Solution quality.

Receiver	Number of Solutions	Fixed Solutions	Float Solutions
Xiaomi Mi 8	595	19 (3.2%)	576 (96.8%)
Stonex S500	617	47 (7.6%)	570 (92.4%)

**Table 2:** Test 2: Post-processing results.

Receiver	RMS E (m)	RMS N (m)	RMS H (m)	STD E (m)	STD N (m)	STD H (m)
Xiaomi Mi8	0.499	0.549	0.556	0.133	0.151	0.411
u-blox ZED F9P	0.021	0.043	0.020	0.179	0.017	0.018

**Table 3:** Comparison between RMS obtained after MDP application, with criterion 1.

Processing	RMS E (m)	RMS N (m)	RMS H (m)	RMS 2D (m)
No MDP	0.450	0.554	0.387	1.428
MDP, crit 1 static thres.	0.427	0.538	0.370	1.374
MDP, crit 1 adaptive thres.	0.410	0.530	0.353	1.339

**Table 4:** Comparison between RMS obtained after MDP application, criterion 1, during multipath-induced interval.

Processing	RMS E (m)	RMS N (m)	RMS H (m)	RMS 2D (m)
No MDP	0.444	0.567	0.384	1.441
MDP, crit 1 static thres.	0.387	0.5393	0.344	1.328
MDP, crit 1 adaptive thres.	0.356	0.519	0.309	1.259

**Table 5:** Comparison between RMS obtained after MDP application, criterion 2.

Processing	RMS E (m)	RMS N (m)	RMS H (m)	RMS 2D (m)
No MDP	0.450	0.554	0.387	1.441
MDP, crit 2, MDP thr = 2.5 m, SNR thr = 20 DB-Hz.	0.448	0.551	0.384	1.424
MDP, crit 2, MDP thr = 2.5 m, SNR thr = 35 DB-Hz.	0.397	0.509	0.333	1.250

**Table 6:** RMS obtained for criterion 1, varying static MDP threshold values.

Processing	RMS E (m)	RMS N (m)	RMS H (m)	RMS 2D (m)
No MDP	0.731	2.075	4.444	4.340
MDP, crit 1, MDP thr = 2.5 m	0.935	1.638	4.063	3.773
MDP, crit 1, MDP thr = 5.0 m	0.696	1.944	3.635	4.129

**Table 7:** RMS obtained for criterion 1, adaptive MDP threshold.

Processing	RMS E (m)	RMS N (m)	RMS H (m)	RMS 2D (m)
No MDP	0.731	2.075	4.444	4.340
MDP, crit 1, adaptive MDP thr	0.867	2.845	3.521	5.949

**Table 8:** RMS obtained for criterion 2, MDP static threshold.

Processing	RMS E (m)	RMS N (m)	RMS H (m)	RMS 2D (m)
No MDP	0.731	2.075	4.444	4.340
MDP, crit 2, MDP thr = 2.5 m, SNR thr = 35 DB-Hz	0.542	1.722	3.929	3.611

## Data Availability

Data used in the experiments will be made available upon request made to the authors.
